# Variation in Tree Species Ability to Capture and Retain Airborne Fine Particulate Matter (PM_2.5_)

**DOI:** 10.1038/s41598-017-03360-1

**Published:** 2017-06-09

**Authors:** Lixin Chen, Chenming Liu, Lu Zhang, Rui Zou, Zhiqiang Zhang

**Affiliations:** College of Soil & Water Conservation, Beijing Forestry University, Qinghua East Road 35, Haidian District, Beijing, 100083 P.R. China

## Abstract

Human health risks caused by PM_2.5_ raise awareness to the role of trees as bio-filters of urban air pollution, but not all species are equally capable of filtering the air. The objectives of this current study were: (1) to determine the foliar traits for effective PM_2.5_-capture and (2) explore species-to-species differences in foliar PM_2.5_-recapture capacity following a rain event. The study concluded that overall, the acicular needle shape made conifers more efficient with PM_2.5_ accumulation and post-rainfall recapture than broadleaved species. The foliar shape and venation of broadleaved species did not appear to influence the PM_2.5_ accumulation. However, the number of the grooves and trichomes of broadleaved species were positively related to foliar PM_2.5_ accumulation, suggesting that they could be used as indicators for the effectiveness of tree PM_2.5_ capture. Furthermore, the amount of PM_2.5_ removal by rainfall was determined by the total foliar PM_2.5_. Not all PM_2.5_ remained on the foliage. In some species, PM_2.5_ was resuspended during the growing season, and thus reduced the net particular accumulation for that species. These findings contribute to a better understanding of tree species potential for reducing PM_2.5_ in urban environments.

## Introduction

PM_2.5_ has raised severe public health concerns as particles easily penetrate the pulmonary alveoli^1^ and pollution issues related to it have become increasingly severe as a result of global climate change. Periodic PM_2.5_ pollution episodes in cities are more likely to develop in winter in the Northern Hemisphere because of increased air temperature as well as more frequent atmospheric inversions under the background of global climate change^2^. Moreover, summer episodes may also increase PM_2.5_ concentrations due to an increased in the likelihood of stationary air masses^3^, intense secondary aerosol formation^4–6^ and forest fires^7–9^. Cessation of vehicular or industrial PM_2.5_ emissions is not economically or functionally practical in highly urban areas such as Beijing. Therefore, cities will have to develop multiple measures to mitigate PM_2.5_ concentrations. Tree planting (a.k.a. “greening”) has been suggested as one method to reduce PM_2.5_ in urban areas because these measures would effectively complement air pollution mitigation^10^. Foliage acts as a bio-filter of air pollution^11^ and improve air quality^12^ due to the leaves’ rough texture and large contact area. Vegetated greenbelts (i.e., areas of natural or planted herbaceous and non-herbaceous vegetation) can effectively reduce the dust and filter the suspended particles that would otherwise impact urban areas^13^. Several previous studies have evaluated the amount of PM_2.5_ removal from urban air by vegetation. For example, concentrations of PM_2.5_ have been shown to decrease by 9% in woodlands immediately adjacent to urban areas^14^. On a larger scale, trees annually removed approximately 300 metric tons of air pollutants from Christchurch, New Zealand^15^. In Beijing, the trees removed 1,261 metric tons of pollutants, 772 metric tons of which was PM_10_^16^. Studies conducted in the UK indicated that planting trees on one-fourth of the available urban area can reduce PM_10_ concentrations by 2 to 10%^17^. Overall, the findings suggest that urban vegetation have a direct and positive effect on human health by reducing PM_2.5_. Thus, tree planting can be considered pollution mitigation measure in a variety of urban settings. However, open space suitable for tree greening programs is limited in cities. Therefore, if urban vegetation is to be employed as a measure for controlling the air pollution, the most efficient species and tree spacing should be used to maximize PM_2.5_ uptake by vegetation. The ability to identify the most efficient vegetation attributes and species for capturing PM_2.5_ is important because that will provide a basis for selecting plants to improve air quality in vulnerable areas.

Unfortunately, current studies on foliar PM_2.5_ capture efficiency are inconclusive. Significant differences have been reported between and within modelling and experimental studies. A number of such studies were conducted to evaluate deposition velocity (V_d_, cm s^−1^)^18^, deposition amount (mg cm^−2^ or μg cm^−2^)^19, 20^, magnetic deposition velocity (cm^−1^)^21^, particle number (mm^−2^)^22^, and particle cover area (%)^23^. For example, PM_10_ deposition velocities (V_d_) on vegetation varied from ~0.01 to ~10 cm s^−1^ ^24^, but models were not very accurate at predicting V_d_ within a species. Simulated V_d_ of 1 μm diameter particles on *Picea abies* were calculated to be 0.02 cm s^−1^ ^25^, but measured rates of V_d_ were 0.55 cm s^−1^ ^26^, a 25-fold difference. Differences between modeled and measured V_d_ can be attributed to uncertainties associated different physical and chemical processes involved in tree-atmosphere interactions such as PM_2.5_ capture model sensitivity to different plant boundary layer parameters, and to the importance of initial conditions^27^. Measured particle V_d_ are not only dependent on the measurement methods^17^ but also on a number of other factors such as particle size and density^28^, concentrations of other pollutant (e.g., ozone)^29^, meteorological conditions (e.g. precipitation affecting particle removal from the leaves^30^ and wind^31^ affecting resuspension and boundary layer heights^32^) and the tree canopy morphology^30^. Although foliar PM_2.5_ deposition varies with *in*-*situ* conditions, the foliar PM_2.5_ accumulation is often just considered to be a species-specific^33^. An examination foliar PM_2.5_ retention ability for different species under similar conditions is necessary to better rank tree species efficiency for capturing PM_2.5_.

Given that the surface properties of objects are known to influence particle immobilization^34^, plant species differ in their ability to scavenge dust-laden air^24, 34^. The dust-retention abilities of vegetation depend on several factors including canopy type, leaf and branch density, and leaf micromorphology (e.g., roughness, trichomes and wax)^18, 19, 31, 33, 35–39^. Conifers are considered to be more effective in PM_10_ capture than broadleaved species^34^ and evergreen conifers have the potential to accumulate pollutants throughout the year. Within the broadleaved species, rough leaf surfaces are more efficient in capturing PM_2.5_ than those with smooth leaf surfaces^31, 40^. Within species cultivar, leaf surface property variation can also impact PM_2.5_ capture. Large-scale sampling must be conducted to quantify the relationships between species traits and PM_2.5_ capturing capacity.

Although it is understood that the temporary retention of particles by urban trees can reduce atmospheric PM_2.5_ concentrations, the effectiveness of vegetation as a long-term alternative to other measures is still under debate^24^. Most particles are retained on the plant surface and subsequently removed from the canopies by resuspension to the atmosphere through rainfall and leaves fallen to the ground^30, 41^. Particle fate is also impacted by other factors, including canopy characteristics, micrometeorological conditions, particle size and leaf morphology^18, 31, 42, 43^. However, to date, no study has systematically compared interspecies effectiveness in capturing and retaining PM_2.5_. It is not applicable to deduce the foliar PM_2.5_ deposition and resuspension from PM_10_ studies due to the weak correlations between the PM size fractions^44^.

Therefore, the aim of this study is to (1) quantify the relationship between PM_2.5_ accumulation and the leaf macromorphology and micromorphology using a large sampling population and (2) explore the differences in PM_2.5_ retention under rainfall conditions by leaves of different species. To our knowledge, this is the first study to quantify the relationship between leaf macromorphology and foliar PM_2.5_ capture. The findings from this study have practical implications for urban tree species selection targeting air pollution. Moreover, the results presented here will assist urban planners to evaluate the potential capacity of PM_2.5_ removal from the long term, large scale perspective.

## Results

### Foliar PM_2.5_ accumulation of tree species

**Figure 1:**
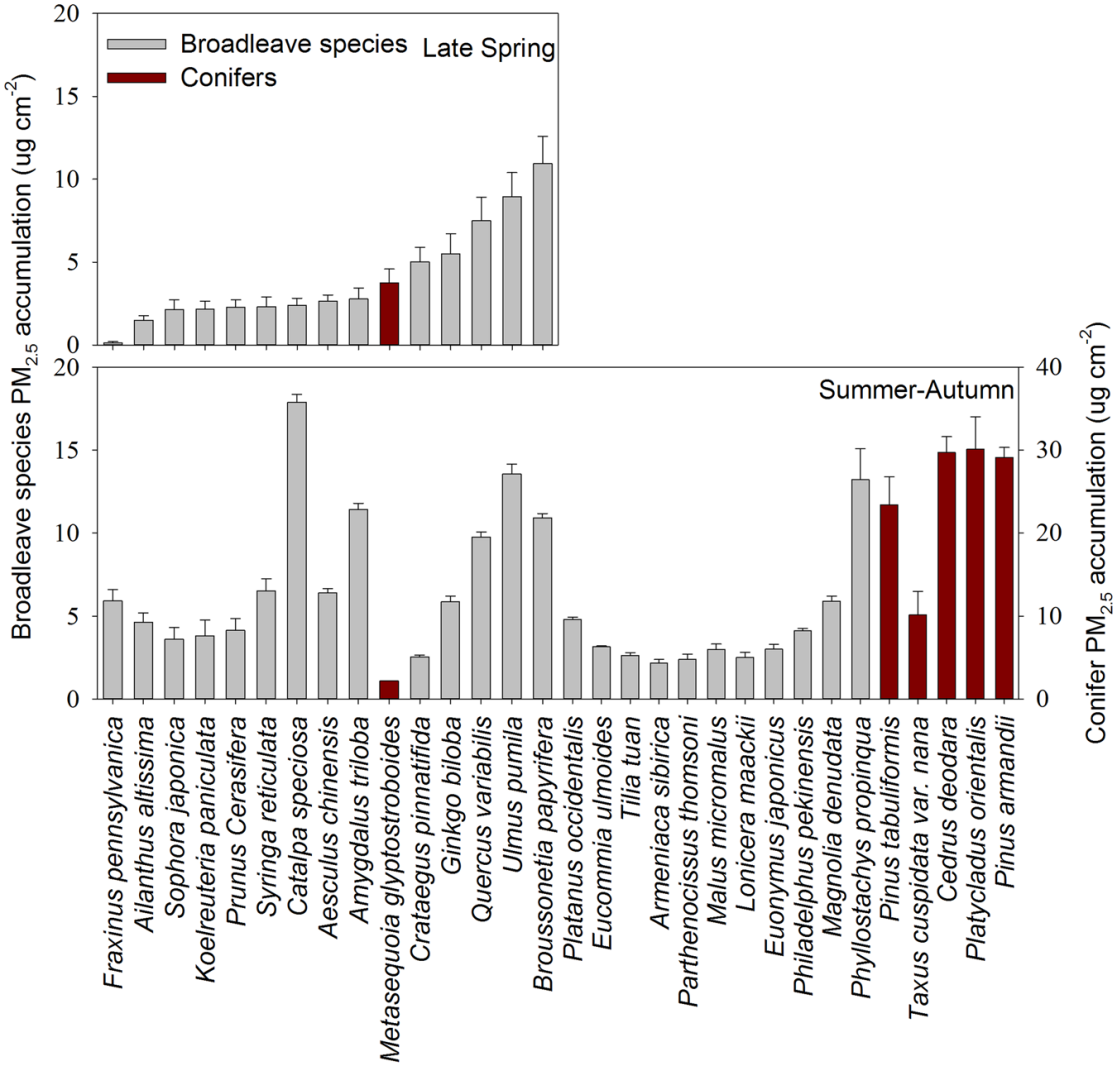
Comparison of foliar accumulation of atmospheric PM_2.5_ among by different tree species (coniferous and broadleaved) measured in (**a**) late spring and (**b**) summer through autumn. The within-sample variability of PM_2.5_ of each species presented as error bars.

Atmospheric PM_2.5_ captured on the leaves of urban trees varied among species and seasons (Fig. 1, see also Supplementary Table S1). Coniferous species predominately contained the largest accumulation (>20 μg cm^−2^) of PM_2.5_ were predominantly coniferous species (Fig. 1b). Conversely, the four most efficient broadleaved PM_2.5_-retention species were *Catalpa specieosa*, *Ulmus pumila*, *Amygdalus triloba and Broussonetta papyrifera*, all characterized by leaves covered with dense hairs, whereas the least effective species were*Tilia tuan*, *Armeniaca sibirica and Lonicera maackii*, which had smooth leave surfaces.

### Tree morphological traits and leaf retention of PM_2.5_

**Figure 2:**
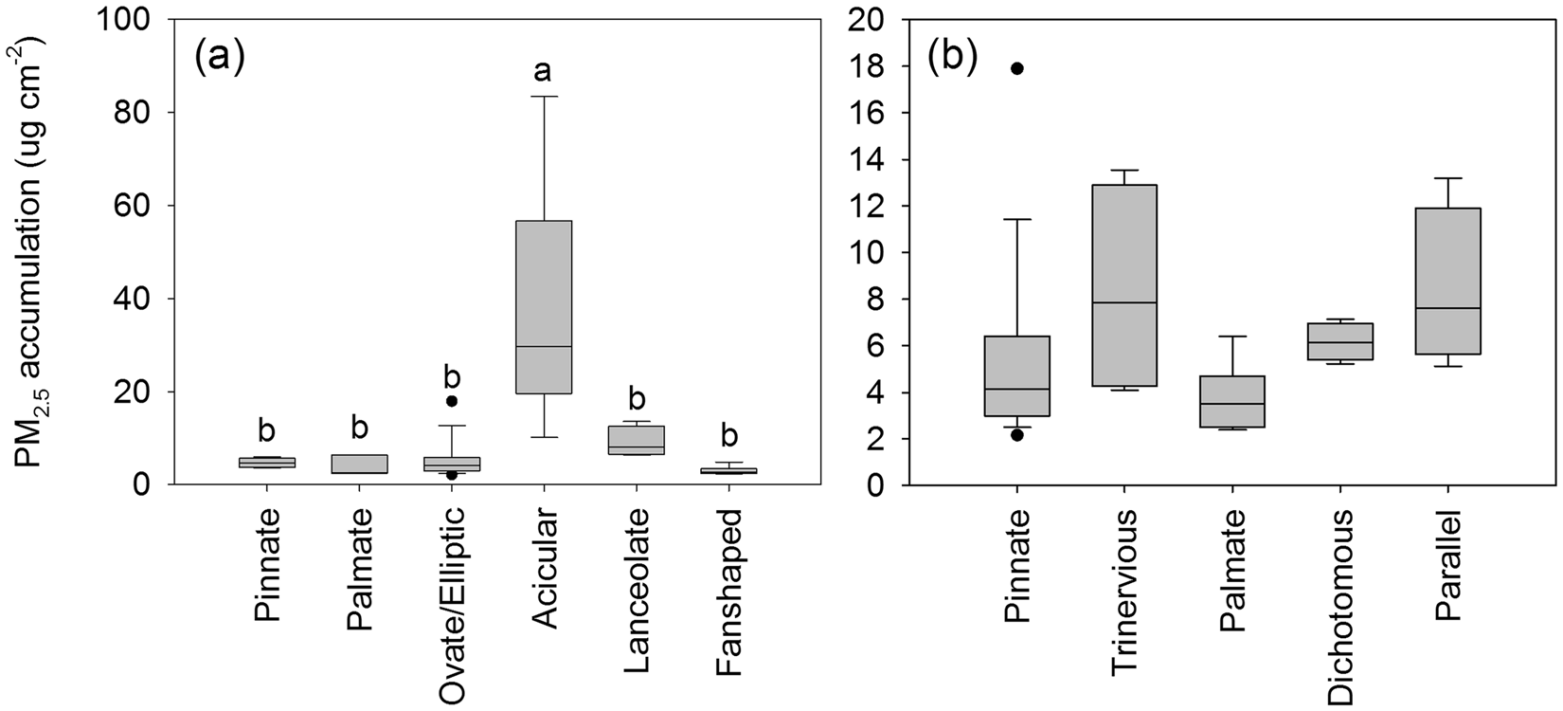
Differences in foliar PM_2.5_ accumulation between different leaf shapes (**a**) and venation patterns (**b**) pooled from all species measured. Bars are means ± SE. Different letters above the error bars indicated significant differences between leaf shapes (Bonferroni test, α = 0.05).

Acicular (needle-shaped) leaves showed the highest capacity to capture PM_2.5_ (one-way ANOVA, P = 0.01, Fig. 2a), followed by lanceolate leaves. No significant differences (P = 0.09) in the PM_2.5_ amount per unit leaf area were observed between the different venation patterns of the studied species, although trinervious veins exhibited favourable PM_2.5_ capturing effect (Fig. 2b).

**Figure 3:**
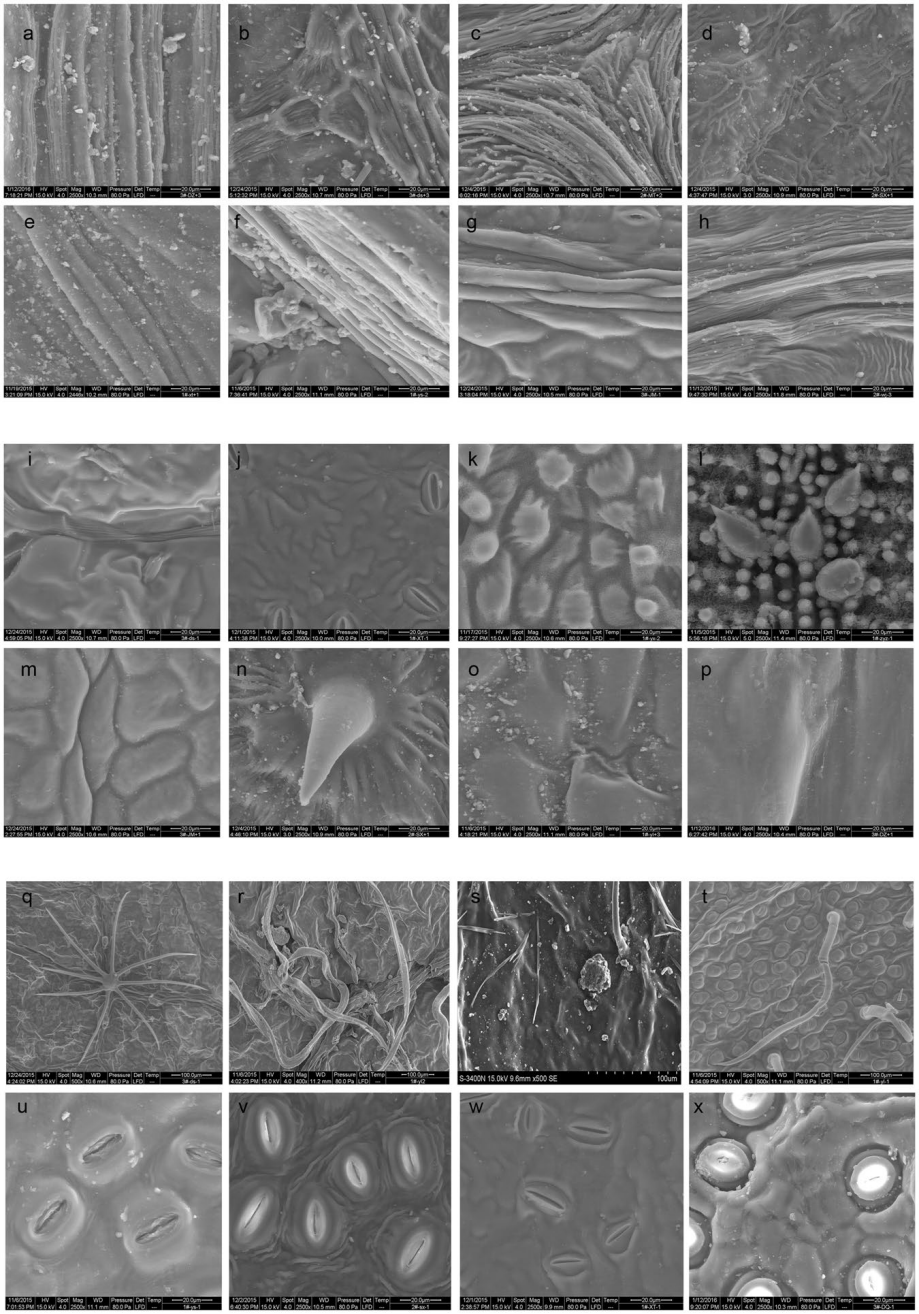
Images of leaf surface micromorphology and deposited particulate matters. The images corresponded to the samples of: (**a**) *Eucommia ulmoides*, (**b**) *Tilia tuan*, (**c**) *Platanus occidentalis*, (**d**) *Armeniaca sibirica*, (**e**) *Malus micromalus*, (**f**) *Ulmus pumila*, (**g**) *Lonicera maackii*, (**h**) *Parthenocissus thomsoni*, (**i**) *Tilia tuan*, (**j**) *Philadelphus pekinensis*, (**k**) *Ginkgo biloba*, (**l**) *Phyllostachys propinqua*, (**m**) *Lonicera maackii*, (**n**) *Armeniaca sibirica*, (**o**) *Magnolia denudate*, (**p**) *Eucommia ulmoides*, (**q**) *Tilia tuan*, (**r**) *Broussonetia papyrifera*, (**s**) *Sophora japonica*, (**t**) *Magnolia denudate*, (**u**) *Ulmus pumila*, (**v**) *Armeniaca sibirica*, (**w**) *Philadelphus pekinensis*, (**x**) *Ilex chinensis*.

Digitized morphological features were extracted from leaf images (Fig. 3). The groove ratio (groove area/total leaf area) ranged from ~3 to 25% across all species. Trichomes are the fine outgrowths (including various types of hairs) or appendages on plants. Among the examined species, 53% had no trichomes, (i.e., trichome density (LH_ave_ = 0). The remaining species had trichomes with varying morphologies and densities including sparsely distributed trichomes (LH_ave_ = 5.6 ± 1.13 mm^−2^) or densely covered trichomes (LH_ave_ = 19.9 ± 5.20 mm^−2^). The stoma densities ranged between 40 and 140 mm^−2^ and diameters ranging between 10 and 25 μm.

Across species, the highest rates of PM_2.5_ capture were observed on foliage with micromorphological structures that included dense grooves (Fig. 3a–h) and epicuticular trichomes (Fig. 3q–t). *Armeniaca sibirica* (Fig. 3n) and *Phyllostachys propinqua* (Fig. 3i) both had pointy, protrusive structures. However, *A*. *sibirica* had grooves around the protrusion and was able to capture more PM_2.5_ than *P*. *propinqua* that lacked these grooves. Protrusions that were flattened were not as efficient at retaining PM_2.5_ (Fig. 3i–p) compared to protrusions that were pointy. However, the presence of stoma did not necessarily lead to a larger capacity for PM_2.5_ capture (Fig. 3u–x).

**Figure 4:**
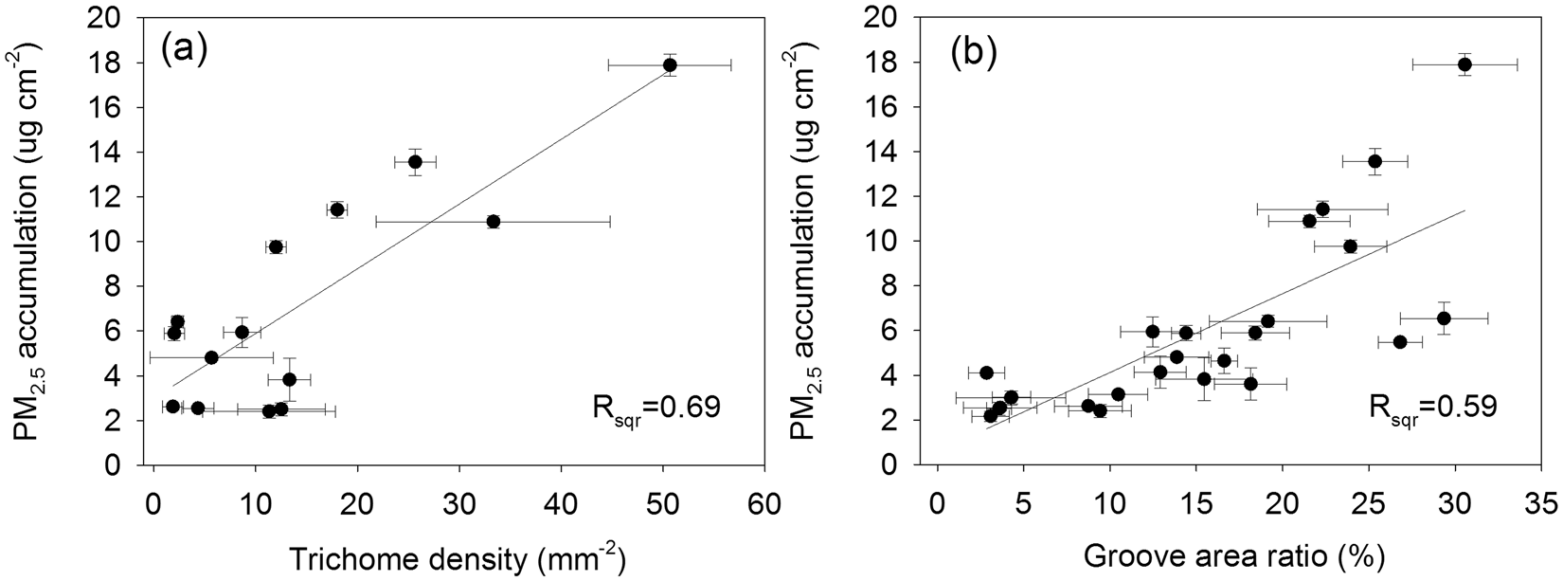
Relationship between foliar PM_2.5_ accumulation and trichome density (**a**) and groove area ratio (**b**).

A correlation analysis indicated that the amount of captured PM_2.5_ trapped particulate matter was positively correlated with the total epicuticular trichomes (R = 0.69, Fig. 4a) and the groove density (Fig. 4b). No relationship was found between foliar PM_2.5_ accumulation and the stomatal density or diameter.

### Removal of PM_2.5_ from leaf surfaces by simulated rainfall

**Figure 5:**
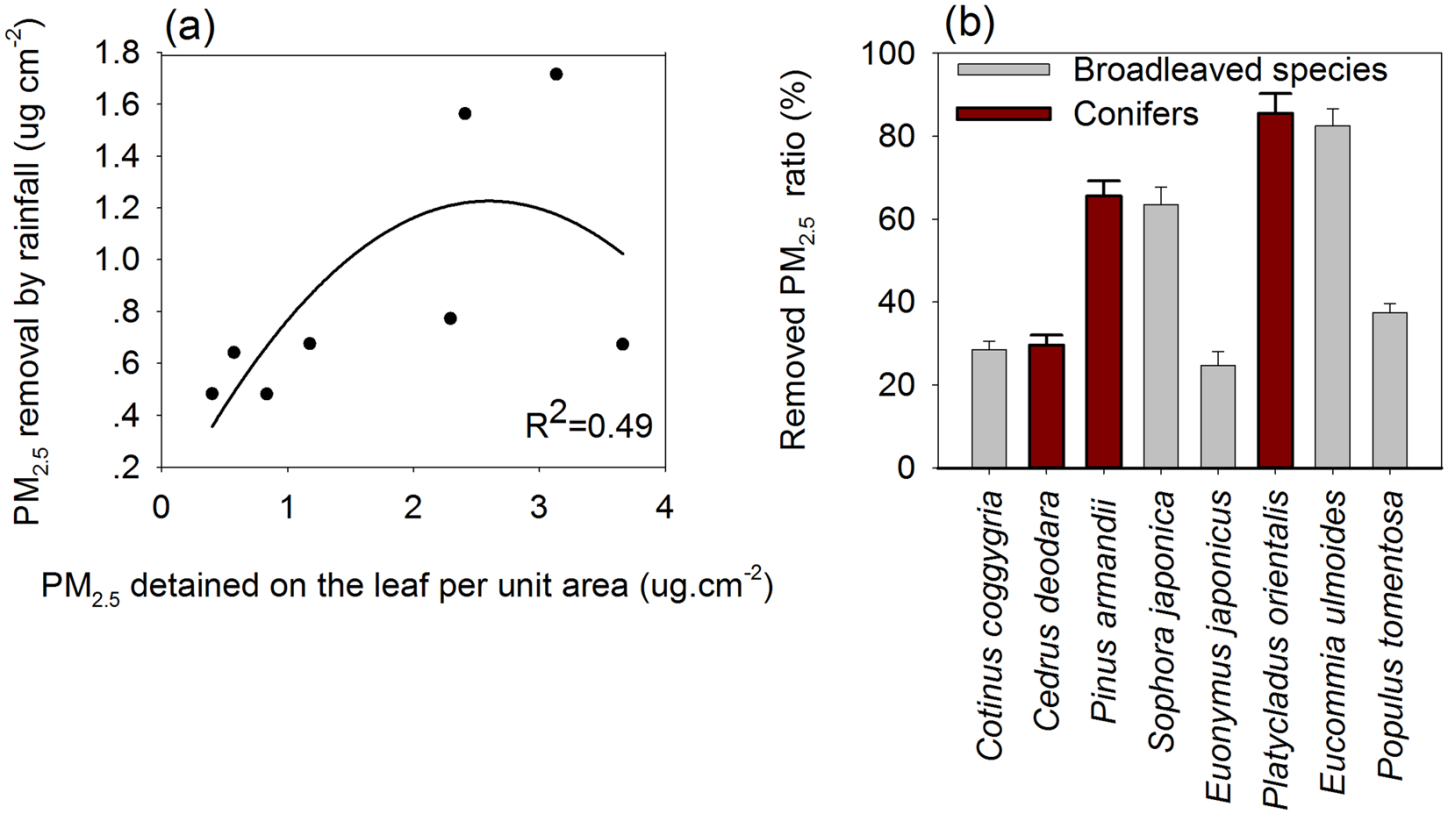
Foliar PM_2.5_ removed by simulated rainfall (equivalent to 7.5 mm outdoor rainfall over 15 minutes) in relationship to total foliar PM_2.5_ accumulation (**a**) and PM_2.5_ removal percentage among species (**b**).

**Figure 6:**
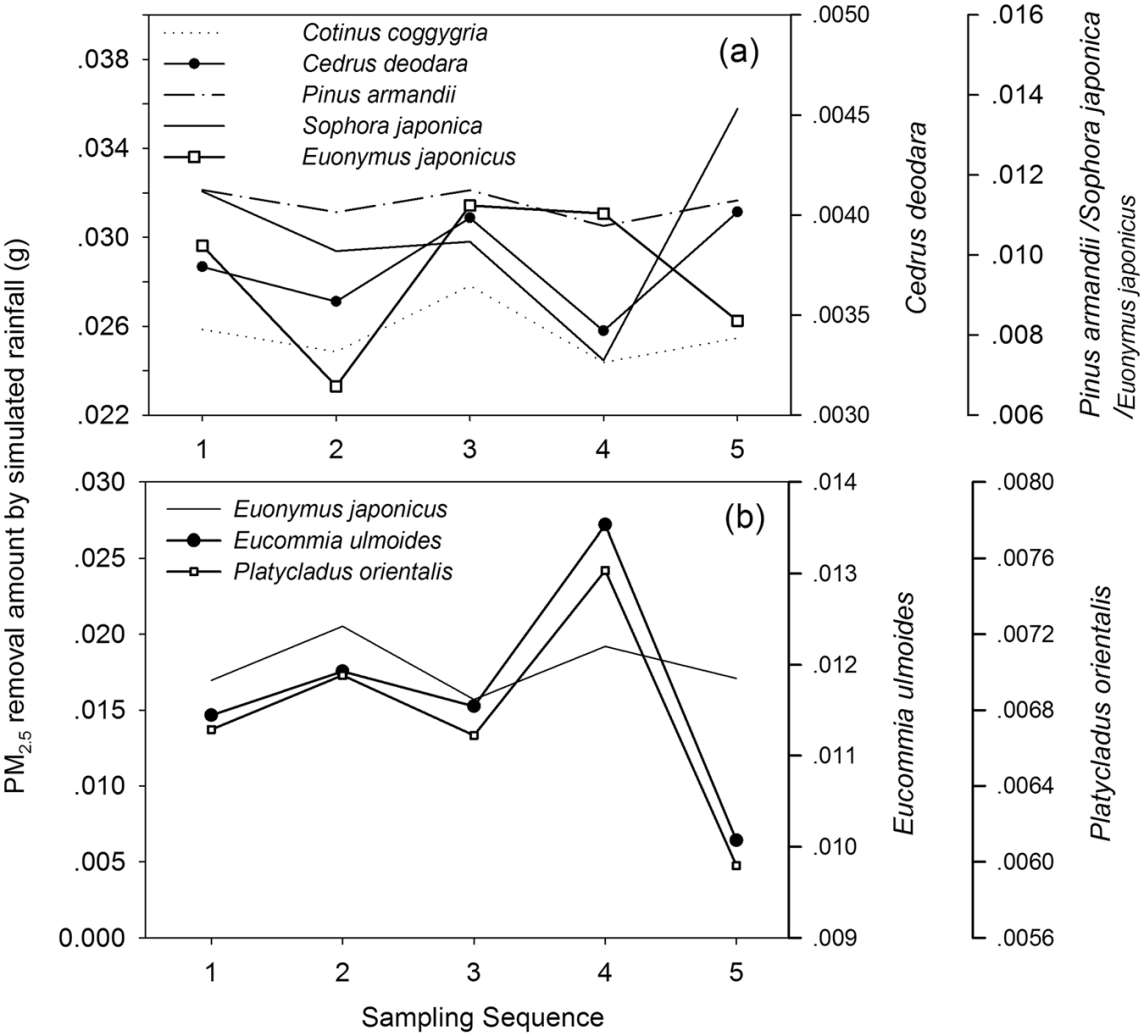
Progression pattern of foliar PM_2.5_ removal by simulated rainfall (equivalent to 7.5 mm outdoor rainfall, over 15 minutes). The sampling time interval was 3 minutes. Sampling sequence from 1 to 5 represented samples taken at 3 minutes, 6 minutes, 9 minutes, 12 minutes and 15 minutes, respectively, from the start of simulated rainfall.

PM_2.5_ removal by rainfall was correlated with the amount of the pollutant retained on the leaf before the rainfall simulation (Fig. 5a). This is in consistent with the fact that rainfall scavenging being a first-order process, and dependent on leaf particle concentration^45^. The PM_2.5_ retention ability of different species varied significantly (one-way ANOVA, P = 0.03) (Fig. 5b). The average removal rate of the foliar PM_2.5_ of the examined coniferous species was 60% (SE = 4%) while the removal rate for broadleaf species was 47% (SE = 3%). *Platyladus orientalis* and *Pinus armandii* lost up to 86% (SE = 5%) and 66% (SE = 4%) of their foliar PM_2.5_, respectively. Only *Cedrus deodara* demonstrated a lower foliar PM_2.5_ removal rate of 30%. Among the broadleaf species, *Eucommia ulmoides* and *Sophora japonica* demonstrated the highest average foliar PM_2.5_ removal rate of 82% (SE = 4%) and 64% (SE = 4%), respectively. The removing process of PM_2.5_ from the leaf surface by rainfall fluctuated with time (Fig. 6). It indicated that the removal process was species specific and subject to rainfall duration and species (Fig. 6).

## Discussion

### Species differences in accumulating PM_2.5_

The influence of morphological traits on foliar PM_2.5_ accumulation was reflected by the changes in the sequence along the temporal progression (Fig. 1). For broadleaf species, large area of foliar ultrastructures, such as grooves, trichomes and glands, were exposed and captured the ambient PM_2.5_ during the process of leaf expansion. The PM_2.5_-capture capacity of such species is expected to increase as they grow mature. This has been observed in *Ginkgo biloba*, *Ulmus pumila*, and *Salix babylonica* ^23^. Therefore, a collection of species with different phenology would maximize the PM_2.5_ trapping effects. Thus, increased biodiversity would extend the period of leaf expansion and maximize PM_2.5_ capture. On a large scale, conifers had higher rates PM_2.5_ compared in broadleaved trees in urban environments^18^. In computer simulation studies, conifers ranked highest in accumulated particulate matter on their foliage^46, 47^. The capture efficiency differences between the needles and the flat leaves can be expressed by the Stoke’s number^31^ which describes the relationship between the stopping distance of a particle and the characteristic dimension of an object (m)^48^. In short, narrow conifer needles have much larger Stoke’s numbers and thus higher capture efficiency. For instance, in the study comparing species including both coniferous and broadleaved trees, the maximum Stoke’s number for the coniferous species was 0.05 while the maximum Stoke’s number for broadleaved species was 0.000012^31^. Additionally, in principle, more turbulent flow could occur across fine cylinders, like coniferous needles, than across large plates, like broadleaves, leading to the reduced boundary layer thickness of needle leaves. Small individual leaf area^49^ of needles is another factor for developing thinner boundary layer in comparison with broad leaves. When the wind carrying PM_2.5_ travels across the leaf, the boundary layer stays relatively stationary and forms a barrier between the surrounding air and the leave surface. Therefore, the thin boundary layer of long narrow needle leaves experienced more potential for PM_2.5_ contact with the leaf surface. Conifer leaf morphology increased the potential for PM_2.5_ capture but did not impact PM_2.5_ release during a rain event as indicated by the high removal ratio (Fig. 5b) under rainfall. Studies on the self-cleaning of leaf surfaces have revealed that epicuticular wax ultrastructures are correlated with the hydrophobic properties of the leaf surfaces and leaf surface PM_2.5_^50, 51^. Therefore, the pine species show greater PM_2.5_ attenuation capabilities in urban areas, especially in winter when pollution concentrations are the highest and broadleaf tree species are leafless. However, pine species are not recommended to use in heavily polluted areas because they are susceptible to pollutant-induced injuries^34, 47^ For example, ozone induces visible injury on pine needles^52^. Sulfur dioxide (SO_2_) causes foliar necrosis in pine trees^53^, and aluminium causes nutrient imbalance and structural changes in the pine needles^54^. Species response to pollutant loading is important for estimating total greening impacts on PM_2.5_ reduction potential. For example, if a conifer has twice the PM_2.5_ capture capacity per unit leaf area of a broadleaf tree, but only a third of the leaf area due to pollutant stress, then the broadleaf species would be more effective at capturing particulate matter at the stand level.

### Influences of leaf micromorphology on PM_2.5_ accumulation

For broadleaved species, leaf shape and venation did not have a significant influence on the PM_2.5_ immobilization because the individual leaves cannot reflect the physical properties of canopy density. Canopy density influences the wind turbulence which has been proposed as a significant explanatory factor for the deposition of particulate matter^55^, especially for the fine particles. Moreover, canopy density has significant influence over the air PM_2.5_ concentration^56^, and thus leads to different PM_2.5_ deposition on leaves. However, the leaf micromorphology such as the groove area and trichomes (Fig. 3) also significantly influences the PM_2.5_ deposition. Foliar surface morphology has been observed to have direct effects on the PM_2.5_ capture by leaves. Specifically, leaf surfaces with grooves or trichomes have a higher capacity for PM_2.5_ retention than smooth leaves (Fig. 4). This finding is consistent with those of previous studies where the degree of leaf roughness and the number of trichomes in upper and lower epidermis of a leaf determined the species dust retention capacity^57, 58^. Additionally, an increased roughness due to leaf hairs, scales, glands, furrows and veins, has been found to increase the particulate accumulation^33, 43, 59–61^. This study also found that dense leaf grooves provided an ideal condition for the deep retention of the PM_2.5_. Foliar trichomes improved the PM_2.5_ capture capacity of leaves. Species with densely haired leaves, such as *Catalpa speciosa*, *Ulmus pumila* and *Broussonetia papyrifera* were the most effective measured species for retaining PM_2.5_ (Fig. 4) as indicated by the amount of PM_2.5_ retention by the leaves after they were fully expanded. The effect of dense trichomes was also reported by other studies. Compared with the adaxial surface, the abaxial leaf surface is less efficient for the deposition of PM_2.5_^61^ due to the lighter micro-roughness of the surface. Therefore, species with abaxial indumentum (a covering of trichomes) were proved more effective in trapping PM_2.5_^31^. For example, the hairy abaxial surface of *Platanus occidental* is reported to be more efficient at capturing PM_2.5_ than the adaxial surface^40^. A previous study^44^ ranked tree species PM_2.5_ capture capacity based on visual observation of trichomes occurrence rather than density as was done in this study. The existence of trichomes would not necessarily correlate with the increased foliar PM_2.5_ retention because sparse hairs had limited PM_2.5_ retention ability. This was consistent with the SEM statistical results that indicated by pattern of PM_2.5_ accumulation for species with a trichome density below 20 mm^−2^ (Fig. 4a). The limited ability of leaves with few trichomes was further evidenced in the SEM images, for species such as *Tilia tuan* (Fig. 3q). With improved trichome statistics from the detailed SEM images in this study, we were able to refine the previous analysis by developing a numerical relationship between trichomes and foliar PM_2.5_ amount. Therefore, visual leaf macromorphological traits may not be sufficient for determining a species PM_2.5_ capture capacity, leaf micromorphological examination may be necessary. This is also the case with the description of foliar roughness represented by groove area ratio.

Stomata size and stomatal density are considered important factors in controlling PM_2.5_ accumulation^62^. For example, an indoor high-dose dust-spray experiment observed discernable particulate distribution around the stomas^35^. However, no significant influence of stomatal charateristics on foliar PM_2.5_ amount trapping was observed in our study. The complex wind turbulence in the outdoor environment may create changing diffusion conditions leading to the lower exposure doses for the plants and disturbances for the deposition of particulate matters. Therefore, the foliar deposition of particulate matters in the controlled experiment would be more evident than in the outdoor natural environment. Moreover, this might be attributed to the lower stomatal density and stomata size in our samples than those in the studies that observed significant relationship between stomatal features and particles. Another study conducted in Beijing^63^ observed that leaves with high stomatal density (>189 mm^−2^) demonstrated significant increase with stomatal density in trapping PM_2.5_. However, this relationship was absent among species with lower stomatal density. Therefore, stomatal density appears to only increase particle capture when the stomatal density is high. This is proved by the comparison of another pair of studies. Stomatal density was related to foliar accumulation of air contaminant for species with high leaf stomatal density (ranging averagely from 237–757 mm^−2^)^64^. By contrast, in another study where the leaf stomatal density ranged from 10.36–38.36 mm^−2^ in average, no significant relationship was found between foliar particle accumulation and stomatal density^65^. Also, the stomatal size in our study was low, ranging from 10 to 25 μm. The study that observed similar stomatal size range (14.5–19.9 μm)^64^ also failed to show consistent relationship between stomata size and foliar air pollutant accumulation. By contrast, this relationship was found in the study where the stomata size ranged from 20 to 192 μm^65^. The state of stomatal opening is another reason for the inconsistent relationship between stomatal density or stomata size and foliar PM_2.5_ accumulation. The stomatal density and stomata size does not necessarily stand for the opening size of the stomas. Sensitive to ambient environmental factors, such as light and water status, the stomas can open to different extent, and thus lead to different rates of transpiration which in turn alters relative humidity. Given that relative humidity influences dry deposition velocity, foliar accumulation of PM_2.5_ capture could be impacted^66^. Although the particles have been observed to enter the leaf through stomatal openings^67^, the frequency to which this occurs is unknown. Blockage of stomata with PM could significantly decreased stomatal conductance and gas exchange, which may further influence the water regime, photosynthesis^67^, and overall plant growth^68^. Therefore, we believe the entering of PM_2.5_ through the stomatal openings should be an occasional observation.

### Rainfall effect on PM_2.5_ retention

The across-seasonal comparisons of the same species indicated that the foliar PM_2.5_ accumulation did not necessarily increase with time (Fig. 1), which may have been caused when the maximum loading capacity of leaves was reached^3, 23, 58^. For example, plant leaves reached their maximum PM_2.5_ loading capacities after 26 days of no rainfall in Guangzhou, China^69^. Therefore, the accumulation of PM_2.5_ on leaves is not linearly related to exposure duration. Foliar PM_2.5_ accumulation is dynamic. Leaves may capture PM_2.5_ for some time before a wind event releases the material back into the air, or a rain event washes the material off of the leaf. Therefore, the amount of foliar PM_2.5_ at the end of the examined period cannot be interpreted as a representation of the total mass of foliar PM_2.5_ accumulation during the corresponding period (e.g., growing season).

This study examined the differences in rainfall removal of foliar PM_2.5_ among species (Fig. 6b). Although the kinetic energy of rainfall is the predominant factor in the foliar washing process^70^, leaf surfaces features like wax layer, trichomes and other protrusions can result in different contact angle between water droplet and different leaf surfaces^71^. These factors create different water-repellent performances between species^72^. Moreover, the hydraulic pressure change due to the impaction of raindrop can change the contact angle and thus the leaf wettability^73^ which could also contribute to the PM_2.5_ rainfall removal patterns. Measurements taken immediately after a rainfall indicated that once deposited, coarse and fine particles were not easily washed off of the leaves^18^. Therefore, modelling the associated processes throughout the entire season to obtain an accurate estimate of the amount of PM_2.5_ immobilized by urban trees is necessary.

The amount of PM_2.5_ accumulated on a leaf is therefore a combination of multiple factors, including species factors and meteorological condition^24^. The amount of washed-off PM_2.5_ was not significant related to the leaf morphological traits but was significantly related to the total foliar PM_2.5_ accumulation. This result suggests that mechanisms that determine rainfall loss of foliar retention of PM_2.5_ may apply equally to broadleaf and coniferous species.

The present study demonstrates the PM_2.5_ attenuation dynamics of different species and provides insights for species selection for PM_2.5_ pollution mitigation in urban areas. Nevertheless, trees may also act as PM_2.5_ sources by emitting biogenic volatile organic compounds (BVOCs)^74^ and enhancing local PM_2.5_ concentration under dense planting schemes^75^. BVOCs can react with nitrogen oxides (NO_x_) to form O_3_ and secondary organic aerosol (SOA)^76^. Therefore, the release of BVOCs from vegetation may pose a problem if the planted species are high emitters^77^. BVOC emissions can vary widely among tree species and even within species^78, 79^, depending on physiological and environmental factors^76^. For instance, in Mediterranean areas, where summer is usually characterized by high temperature and little precipitations, the potential for BVOC and ozone formation is high^78^. Therefore, BVOC emissions should be considered during the design of urban green spaces and trees with high BVOC emissions should be avoided to achieve the improved net air pollution reduction benefits from the tree planting. Although PM_2.5_ dispersion was not the focus in the present study, the release of deposited PM_2.5_ from foliage should be considered. A simulation of tree and shrub effects on particle dispersion suggested that particulate matter concentrations would be highest on streets with a high density of trees^80^. Based on dispersion conditions, different planting configurations were showed to have varied abilities to mitigate airborne PM_2.5_ concentrations^44^. Therefore, to optimize the benefits of trees in various urban settings, the pros and cons of different taxa in relation to PM_2.5_ pollution must be considered^81^.

In conclusion, needle-leaved coniferous species are more efficient at removing atmospheric PM_2.5_ and have a higher potential than broadleaved species to recapture PM_2.5_ after rain events. For broadleaved species, macromorphological traits, such as leaf shape and venation, do not have a significant influence on foliar PM_2.5_ retention, whereas micromorphological traits, such as grooves and trichomes, are strongly correlated with foliar PM_2.5_ accumulation and can be used as effective species selection criteria. The temporal process of foliar PM_2.5_ wash-off is highly species-specific, which implies the influence of rainfall duration and intensity on the ability of leaves to accumulate PM_2.5_. A cross-season comparison showed positive and negative increases in PM_2.5_, which reflected the varying influence of resuspension on the amount of foliar PM_2.5_ accumulation. These findings indicate that the accumulation-suspension cycle of urban trees must be further investigated to accurately evaluate accurately the long-term potential bio-filtration capacity of different trees.

## Methods and Materials

### Leaf-washing experiments for the species comparison

Samples for the leaf-washing experiments were collected on the campus of Beijing Forestry University (40°00′ N, 116°34′ E), Beijing, China, which offers abundant vegetation species within a radius of 500 m. Thus, it is reasonable to assume (for the purpose of comparison) that the vegetation is exposed to the same PM_2.5_ concentrations. Thirty-one tree species (specific species are listed in the results) were tested for their capacity to accumulate PM_2.5_ on the leaf surface. Leaves of 15 species were collected in April 2015 as late spring samples, and the leaves of 16 additional species were collected during September 2015 as the summer-autumn samples. The following criteria applied to leaf collection days: sunny and wind speed less than 5 m s^−1^. Lower wind speeds ensured that the wind would not affect the particle deposition on the leaf ^82^. A portable meteorological station (NK4500, Kestrel Co., Philadelphia, PA, USA) was used to measure the sampling conditions in the middle of an open green space at a height of 10 m. All the branches were firmly held and cut carefully from a height of 1.5–2.5 m to avoid losing particulate matter from the leaves. Each species included three sampled trees, and eight small peripheral branches at four azimuth angles were cut from one sampled tree. This sample size provided a leaf area range of 300 to 500 cm^2^. During collection, samples were immediately closed and labeled in plastic bags to avoid contamination, and they were stored in the lab in a freezer (−18 °C).

PM_2.5_ can be trapped both on the leaf surface and in the leaf wax. However, only PM_2.5_ deposition on the leaf surface was assessed in this study because of the environmental concerns of using chloroform^81^ and the relatively low ratio of in-wax PM_2.5_^20^. Once the samples were removed from the freezer, 10 g (fresh weight) of leaves were washed with distilled water and brushed carefully so that the PM_2.5_ was fully removed from the leaf surface. The solution was run through a metal sieve with a mesh diameter of 100 μm to obtain a suspension of liquid sample I. Ten percent of the liquid sample I was injected into pre-weighted PP plastic bag (W_1_) and dried. The dried bag was weighted again (W_2_), and the difference between W_2_ and W_1_ was the weight of 10% of the total suspended particulates (TSP) in the rinse water, which was translated to the TSP amount (W_TSP_) in the original liquid sample by dividing by 0.1. The remaining 90% of water sample I was pumped through filters (PTFE membrane, Whatman, UK) of 10 μm and then of 2.5 μm to intercept particles with a diameter of 10–100 μm and 2.5–10 μm, respectively^81^. The filters used for the analysis were first soaked in distilled water for 2 hours and then dried at 105 °C in a drying chamber for 3 hours to remove soluble impurities, and they were then placed in a balancing chamber for 48 hours to stabilize the humidity change. Filters were weighed before and after filtration (XS105DU balance, Mettler-Toledo International Inc., Switzerland). The resulting weight of the PM_10-100_ and PM_2.5-10_ only account for 90% of the original rinsing liquid and therefore should be divided by 0.9 to obtain the total PM_10-100_ (${{\rm{W}}}_{{\text{PM}}_{10 \mbox{-} 100}}$) and PM_2.5-10_ (${{\rm{W}}}_{{\text{PM}}_{2.5 \mbox{-} 10}}$). The PM_2.5_ mass was then calculated as the difference between the W_TSP_ and the sum of ${{\rm{W}}}_{{\text{PM}}_{10 \mbox{-} 100}}$ and ${{\rm{W}}}_{{\text{PM}}_{2.5 \mbox{-} 10}}$.

To facilitate the species comparison, leaf area-normalized PM_2.5_ accumulation results (i.e. in the unit of μg cm^−2^) are required. Therefore, the leaves were scanned, and the surface areas were obtained from digital images processed with Photoshop (version: Photoshop CS5, Adobe Systems Incorporated, San Jose, CA, U.S.). Branches were excluded from the PM_2.5_ deposition quantification due to issues with accurately assessing branch area and the potentially low ratio in the total plant area index^83^. For needle leaves, we measured water displacement to determine leaf volume and converted the volume to the leaf area according to the following formula:

1 $${A}_{L}=2L(1+\frac{\pi }{n})\sqrt{\frac{nV}{\pi L}}$$

where *A*_*L*_ is the leaf area, *V* is the water displacement volume as a the substitute of the needle-leaf-volume, *n* is the number of needle leaves in a single bundle, and *L* is the average length of the needle leaves^84^.

### Microscopic observation of foliar morphology

Leaf samples were examined under an environmental scanning electron microscope (ESEM, Quanta 200 FEG, FEI, USA) operated in the low vacuum mode (15 kV, 80 Pa) to test for relationships between foliar micromorphological traits and PM_2.5_ accumulation. These analyses were conducted on the same day to prevent desiccation and subsequent alteration of leaf surface micromorphology^3^. Two pieces (1 × 1 cm^2^) were excised from the centre of the lamina of each leaf ^22^. Then, two adaxial specimens and two abaxial specimens^23^ were coated with a thin conductive film of platinum in order to increase electrical conductivity and improve optical transmission. The processed samples were mounted on the stubs for microscopic observations. Grooves, trichomes and stomata were included for further analysis after a preliminary visual screening of the scanning images to determine the effective micromorphological traits for PM_2.5_ retention. The roughness of the leaves on the abaxial or adaxial side was quantified in terms of the groove area ratio (%), which represents the ratio of groove area to total leaf area of the specimen;

2 $${Groove}\,{area}\,{ratio}=\frac{{A}_{G}}{{A}_{L}}\times 100 \% $$

where *A*_*G*_ stands for groove area (μm^2^) and *A*_*L*_ stands for total leaf area (μm^2^). Due to the overlapping of trichomes, this trait could not be quantified in a measurable unit. Instead, the pubescent area (defined as the surface bearing trichomes) as calculated as a measurement of the trichomes density. The stomata were quantified in terms of stomatal density (SD, mm^−2^) and stomatal size (evaluated in diameter, DS, μm).

### Simulated rain wash experiment

A controlled rainfall wash experiment was conducted to evaluate the species differences in resisting rainfall removal of foliar PM_2.5_. Branches with leaves of different species were fixed in a container with a hole at the bottom for sampling water, and they were then placed underneath an artificial rainfall system. The system was installed at the ceiling of a room with a base area of 400 m^2^. Based on the statistical data provided by Haidian District Water Authority, Beijing, China (http://hdsw.bjhd.gov.cn/zxfw/bmcxfw/hdqyqcx/index_3.htm), the average daily rainfall during the sampling period was 8.4 mm, and individual rainfall event s varied between 2–10 mm most frequently in the region. Combined with the settings of the system, the samples were subjected to a simulated rain event of 7.5 mm that lasted for 15 minutes. The water containing the washed foliar PM_2.5_ was sampled every 3 minutes to examine the change in the amount of PM_2.5_ washed off the leaves. Each species had three repetitions. In addition, to exclude the influence of impurities from the water, a parallel experiment was conducted for the control group, and it included three containers with the same configurations as all of the others but with no branch sample. The final result of the washed-off PM_2.5_ was calculated as the difference between the water samples from planting containers and that from the control group.

### Statistical analysis

Significant differences among species were examined via one-way ANOVA in SPSS 18.0 (SPSS Inc., Chicago, IL, USA). The Bonferroni correction method was applied for the comparison statement because of the unequal sample size among groups. Curve fitting and plotting were conducted using Sigmaplot software 12.5 (Systat Software, San Jose, CA, USA).

## Electronic supplementary material

Supplementary Information
