# Supplementary material for: Variation in Tree Species Ability to Capture and Retain Airborne Fine Particulate Matter (PM2.5)
Source: Sci Rep. 2017 Jun 9;7:3206. doi: 10.1038/s41598-017-03360-1 (PMC5466687; doi:10.1038/s41598-017-03360-1)
Supplement: Supplementary file 1 — Supplementary Information [file 41598_2017_3360_MOESM1_ESM.pdf]

Variation in Tree Species Ability to Capture and Retain Airborne Fine Particulate Matter ($PM_{2.5}$)

Lixin Chen[1,a], Chenming Liu[2,a], Lu Zhang[3,a], Rui Zou [a], Zhiqiang Zhang[a*]

[a] College of Soil & Water Conservation, Beijing Forestry University, Qinghua East Road 35, Haidian District, Beijing 100083, PR China

1,2, and 3 equally contributed to this work;

*Corresponding author Email address: zhqzhang@bjfu.edu.cn.

Species $PM_{2.5}$ accumulation & S.D. ($ug.cm^{-2}$)

	Summer-Autumn	Spring	Summer-Autumn S.D	Spring S.D.
Fraxinus pennsylvanica	5.935	0.134	0.672	0.078
Ailanthus altissima	4.635	1.509	0.566	0.274
Sophora japonica	3.603	2.147	0.720	0.587
Koelreuteria paniculata	3.819	2.166	0.964	0.490
Prunus Cerasifera	4.144	2.278	0.724	0.463
Syringa reticulata	6.539	2.320	0.717	0.579
Catalpa speciosa	17.894	2.399	0.483	0.424
Aesculus chinensis	6.412	2.655	0.257	0.367
Amygdalus triloba	11.424	2.783	0.363	0.660
Metasequoia glyptostroboides	3.748	0.849	5.469	0.044
Crataegus pinnatifida	2.545	5.016	0.120	0.878
Ginkgo biloba	5.881	5.501	0.343	1.217
Quercus variabilis	9.755	7.503	0.302	1.407
Ulmus pumila	13.553	8.947	0.596	1.451
Broussonetia papyrifera	10.893	10.938	0.273	1.667
Pinus armandii	4.801		0.129	
Platanus occidentalis	3.135		0.053	
Eucommia ulmoides	2.623		0.151	
Tilia tuan	2.159		0.222	
Armeniaca sibirica	2.398		0.297	
Parthenocissus thomsoni	2.983		0.324	
Malus micromalus	2.499		0.299	
Lonicera maackii	3.007		0.289	
Euonymus japonicus	4.097		0.140	
Philadelphus pekinensis	5.888		0.310	
Magnolia denudata	13.191		1.900	
Pinus tabuliformis	83.385		3.360	
Taxus cuspidatavar.nana	10.182		2.800	
Cedrus deodara	29.690		1.920	
Platycladus orientalis	30.111		3.901	
Pinus armandii	29.091		1.220	

Weights of PM fractions

**Spring($ug.cm^{-2}$)**	TSP	PM>10	$PM_{2.5-10}$	$PM_{2.5}$
Fraxinus pennsylvanica	30.480	30.247	0.097	0.134
Ailanthus altissima	27.915	17.628	7.890	1.509
Sophora japonica	15.352	6.102	7.103	2.146
Koelreuteria paniculata	54.390	51.454	0.770	2.166
Prunus Cerasifera	74.641	64.115	8.248	2.278
Syringa reticulata	48.638	43.625	2.694	2.320
Catalpa speciosa	30.028	43.625	5.998	2.399
Aesculus chinensis	53.114	40.577	9.883	2.655
Amygdalus triloba	58.748	40.627	15.338	2.783
Metasequoia glyptostroboides	53.531	49.015	0.767	0.849
Crataegus pinnatifida	74.081	47.832	21.234	5.016
Ginkgo biloba	47.419	39.402	2.515	5.501
Quercus variabilis	117.336	100.582	9.251	7.503
Ulmus pumila	43.224	28.735	5.542	8.947
Broussonetia papyrifera	122.446	99.301	12.208	10.938

**Summer-autumn($ug.cm^{-2}$)**				
Fraxinus pennsylvanica	11.873	5.407	0.531	5.935
Ailanthus altissima	21.106	16.239	0.232	4.635
Sophora japonica	9.904	6.044	0.258	3.603
Koelreuteria paniculata	10.481	6.311	0.352	3.819
Prunus Cerasifera	9.439	5.072	0.223	4.144
Syringa reticulata	12.257	5.342	0.376	6.539
Catalpa speciosa	24.748	6.743	0.111	17.894
Aesculus chinensis	14.850	8.407	0.031	6.412
Amygdalus triloba	20.847	9.141	0.282	11.424
Metasequoia glyptostroboides	17.990	11.698	2.543	3.748
Crataegus pinnatifida	71.570	47.812	21.214	2.545
Ginkgo biloba	10.068	3.840	0.347	5.881
Quercus variabilis	23.202	13.334	0.114	9.755
Ulmus pumila	26.533	12.659	0.322	13.553
Broussonetia papyrifera	24.310	13.164	0.253	10.893
Pinus armandii	36.497	5.847	25.848	4.801
Platanus occidentalis	14.017	10.263	0.619	3.135
Eucommia ulmoides	32.504	21.191	8.690	2.623
Tilia tuan	12.767	8.528	2.081	2.159
Armeniaca sibirica	6.814	4.065	0.352	2.398
Parthenocissus thomsoni	67.843	59.973	4.887	2.983
Malus micromalus	7.161	4.382	0.280	2.499
Lonicera maackii	12.787	9.234	0.546	3.007
Euonymus japonicus	26.158	18.555	3.506	4.097
Philadelphus pekinensis	17.754	11.416	0.450	5.888
Magnolia denudata	29.014	14.091	1.733	13.191
Pinus tabuliformis	130.276	103.373	3.518	23.385
Taxus cuspidatavar.nana	18.491	7.796	0.513	10.182
Cedrus deodara	57.465	18.555	9.221	29.690

Platycladus orientalis	55.727	21.631	3.985	30.111
Pinus armandii	57.776	23.459	5.226	29.091
